# Lightweight carbon-red mud hybrid foam toward fire-resistant and efficient shield against electromagnetic interference

**DOI:** 10.1038/s41598-020-66929-3

**Published:** 2020-06-18

**Authors:** Rajeev Kumar, Anushi Sharma, Ashutosh Pandey, Anisha Chaudhary, Neeraj Dwivedi, Muhamed Shafeeq M, D. P. Mondal, A. K. Srivastava

**Affiliations:** 1 CSIR-Advanced Materials and Processes Research Institute, Bhopal, 462026 India; 2 Department of Physics and Astrophysics, University of Delhi, Delhi, 110007 India

**Keywords:** Materials for energy and catalysis, Structural materials

## Abstract

Lightweight, porous, high-performance electromagnetic interference (EMI) shielding and fire-resistant materials are highly demanded in aerospace and defense applications. Due to the lightweight, open porosity and high surface area, carbon foam has been considered as one of the most promising candidates for EMI shielding applications. In the present investigation, we demonstrate the development of novel carbon-red mud hybrid foams with excellent EMI shielding effectiveness (SE). The carbon-red mud hybrid foams are prepared using phenolic resin as a carbon source and red mud (industrial waste) as filler. We observed that the inclusion of red mud in carbon-red mud hybrid foams significantly enhances their dielectric, magnetic, EMI shielding and thermal properties. The EMI shielding results show that absorption is the main contributor to the total EMI SE. The maximum total EMI shielding effectiveness is achieved to be 51.4 dB in the frequency range of 8.2–12.4 GHz for carbon-red mud hybrid foam having 20 wt. % of red mud. The CF-RM20 also showed excellent fire resistance and high thermal stability at elevated temperatures.

## Introduction

In recent time, the rapid development of wireless communication technology and electronic devices such as mobile, laptop, household appliances, medical devices, military radars, and aerospace communications makes our life comfortable and easier^1,2^. However, the generation of undesirable electromagnetic (EM) radiations has an adverse effect which leads to the degradable reliability of highly sensitive electronic equipment as well as the harmful effect on human health^3^. The use of EMI shielding materials is the most effective way to address the above-mentioned problems^4–7^. These materials must have high electrical conductivity to minimize the transmission of EM wave and good EM wave absorbing capability. The metal-based materials are commonly used for EMI shielding due to their excellent electrical conductivity; however, the corrosion susceptibility and high density restrict their further applications in aerospace^8,9^. To resolve these problems, it is extremely important to develop lightweight, highly conducting, corrosion-resistant and high-performance EMI shielding materials. In recent years, porous carbon materials, specially carbon foams, have been widely used in various fields and attracted great attention for EMI shielding applications because of their unique three-dimensional open-cell structure, high surface area, and excellent EM wave absorption^10–14^. It is also observed that different conducting filler contents has been incorporated in the carbon foam matrix for improving the total EMI shielding. Carbon-based fillers, specially natural graphite^4^, carbon fibers^15^, carbon nanotubes^16^, graphene oxide, and graphene^12,17^ with high electrical conductivity and high surface area, have been used to achieve high EMI shielding. Generally, fillers with both dielectric loss and magnetic loss are essential for high EMI shielding with good absorption. However, these conducting fillers can only increase the conductivity and provide dielectric loss in a carbon matrix, thus have a poor effect on EM wave absorption. Additionally, these fillers are very costly and difficult to produce at the bulk scale due to poor dispersion and require purification, auxiliary treatment, and functionalization.

Over the years, metal oxides (Fe_3_O_4_, Fe_2_O_3_, TiO_2_, SiO_2_, Al_2_O_3_, NiO, ZnO and MnO_2_) have also attracted tremendous attention for EMI shielding application. Due to the extraordinary properties of metal oxides, the improvement in EMI shielding and absorption response of these carbon-metal oxide hybrid materials have been reported^18–24^. However, the process for the synthesis of metal oxides is very complex and they are costly which opens the possibility to search a simple and cost-effective solution for the development of carbon-metal oxides composite foams. Nowadays, a huge amount of industrial byproducts or wastes such as fly ash^25^ blast furnace slag^26^, rice husk ash^27^, waste glass^28^, and red mud^29,30^ becoming a serious concern for increasing environmental pollution and generation of a huge amount of unutilized resources. Red mud is one of the industrial wastes produced during the manufacturing of alumina. Production of 1 ton of alumina generates 1–1.5 tons of red mud^31^ hence a large quantity of red mud (about 120 million tons) is produced annually worldwide. Therefore, it is very essential to utilize this red mud for the development of advanced and novel products. Red mud can be used in carbon foam, which may improve the EMI shielding performance and microwave absorption in carbon foam because it contains a different type of metal oxides such as Fe_2_O_3_, Al_2_O_3_, SiO_2_, TiO_2_, CaO, MgO and NaO^29^. Furthermore, these metal oxides (Fe_2_O_3_, Al_2_O_3_, SiO_2,_ and TiO_2_) show excellent microwave absorption property due to the large dielectric and magnetic losses. Up to now, only a few studies were conducted to explore the effect of red mud and clay on the EMI shielding performance of carbon and polymer matrix composites^32,33^. But to the best of our knowledge, utilization of red mud in porous carbon materials or carbon foam for EMI shielding applications has not yet been explored.

In the present study, carbon-red mud hybrid foams with improved EMI shielding and fire-retardant performances are fabricated from phenolic resin as a carbon source and red mud (industrial waste) as a filler using PU foam template method. The EMI shielding performance of carbon-red mud hybrid foams is systematically investigated in X-band (8.2–12.4 GHz). The incorporation of red mud in carbon foam has drastically improved the EMI shielding especially the absorption component and the fire-resistant properties while retaining low density of carbon foam. Additionally, the open-cell structures of the carbon-red mud hybrid foam promote a higher EM wave absorption attenuation. Therefore, the EMI shielding effectiveness of the carbon-red mud hybrid foam has  reached up to 51.4 dB, which is higher than that of pure carbon foam (22.6 dB). Furthermore, the lightweight carbon-red mud hybrid foam gives rise to superior EMI shielding and fire-resistant properties indicating its promising and potential application in defense and aerospace.

## Materials and Method

Phenolic resin supplied by Noida Polychem Pvt. Ltd. Noida Uttar Pradesh, India and red mud from HINDALCO, Renukoot (U.P) were used in this study. Acetone (Molychem, AR grade, 99.5%) was purchased from Channel Ten, T.T. Nagar, Bhopal, (M.P), India. Polyurethane foam was procured from D.D. Enterprises, Bhopal.

**Figure 1:**
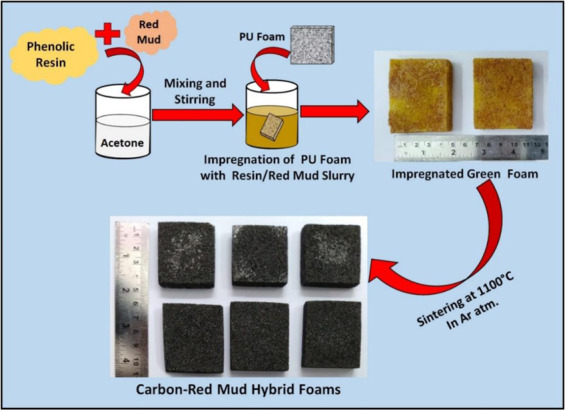
The schematic diagram for the fabrication of carbon-red mud hybrid foams.

In this study, phenolic resin was used as a carbon source and different weight proportions (viz. 0, 5, 10, 15 and 20 wt. %) of red mud particles were used as filler. The carbon-red mud hybrid foams were prepared using PU foam as template^34^. First, the solution was prepared from phenolic resin and acetone with a mass ratio of 30:70 and stirring for 1 hr. Red mud particles were dispersed in acetone for 1 hr. After that dispersed red mud particles were added to phenolic resin solution followed by stirring for 1 hr to make a homogenous slurry of phenolic resin and red mud. The PU foam pieces (size 40 × 40 × 10 mm^3^) were dipped in the homogenous slurry of phenolic resin and red mud under continuous magnetic stirring to obtain phenolic resin and red mud impregnated foam. Impregnated foams were dried at 80 °C for 12 hrs. The dried impregnated foams were cured at 220 °C in the presence of an air atmosphere for 10 hrs for increasing the cross-linking between the polymeric chains. These cured foams were carbonized at 1100 °C for 1 hr in an inert atmosphere to get carbon-red mud hybrid foams. The carbon foam without red mud is designated as CF. For 5, 10, 15 and 20 wt. % of red mud incorporated foams are referred as s CF-RM5, CF-RM10, CF-RM15, and CF-RM20 respectively. The fabrication of carbon-red mud hybrid foams is represented through a schematic in Fig. 1.

### Characterization

X-ray diffractometer (Bruker D8 Advance) with Cu Kα radiation (λ = 0.15418 nm) was used to identifying phases of red mud and hybrid foams in the two theta range of 10–80°. The Raman spectrometer (model: Airix Corporation, Japan) with a laser source 514 nm was used to investigate the defects in red mud and foam samples^35^. The morphologies were determined by field emission scanning electron microscope (NOVA NANO SEM 430). The magnetization of red mud and carbon-red mud hybrid foams was recorded by vibrating sample magnetometer (VSM) (model 7304, Lakeshore Cryotronics Inc., USA) with a maximum magnetic field of 1.2 T, using a perspex holder vibrating horizontally at a frequency of 76 Hz. The compressive deformation behavior of foam samples was tested using a universal testing machine (Instron 8801). The electrical conductivity of foam samples was measured by a d.c. four-probe contact method. The EMI shielding effectiveness was measured in X-band (8.2–12.4 GHz) using a vector network analyzer (VNA E8263B Agilent Technologies). The carbon foam samples with a rectangular size of 26.8 mm × 13.5 mm × 2.0 mm were used for EMI shielding analysis^10^. The thermogravimetric analyzer (Mettler Toledo TGA/SDTA 851^E^) was used to investigate the thermal stability of hybrid foams in the air at a heating rate of 10 °C/min.

## Results and Discussions

**Figure 2:**
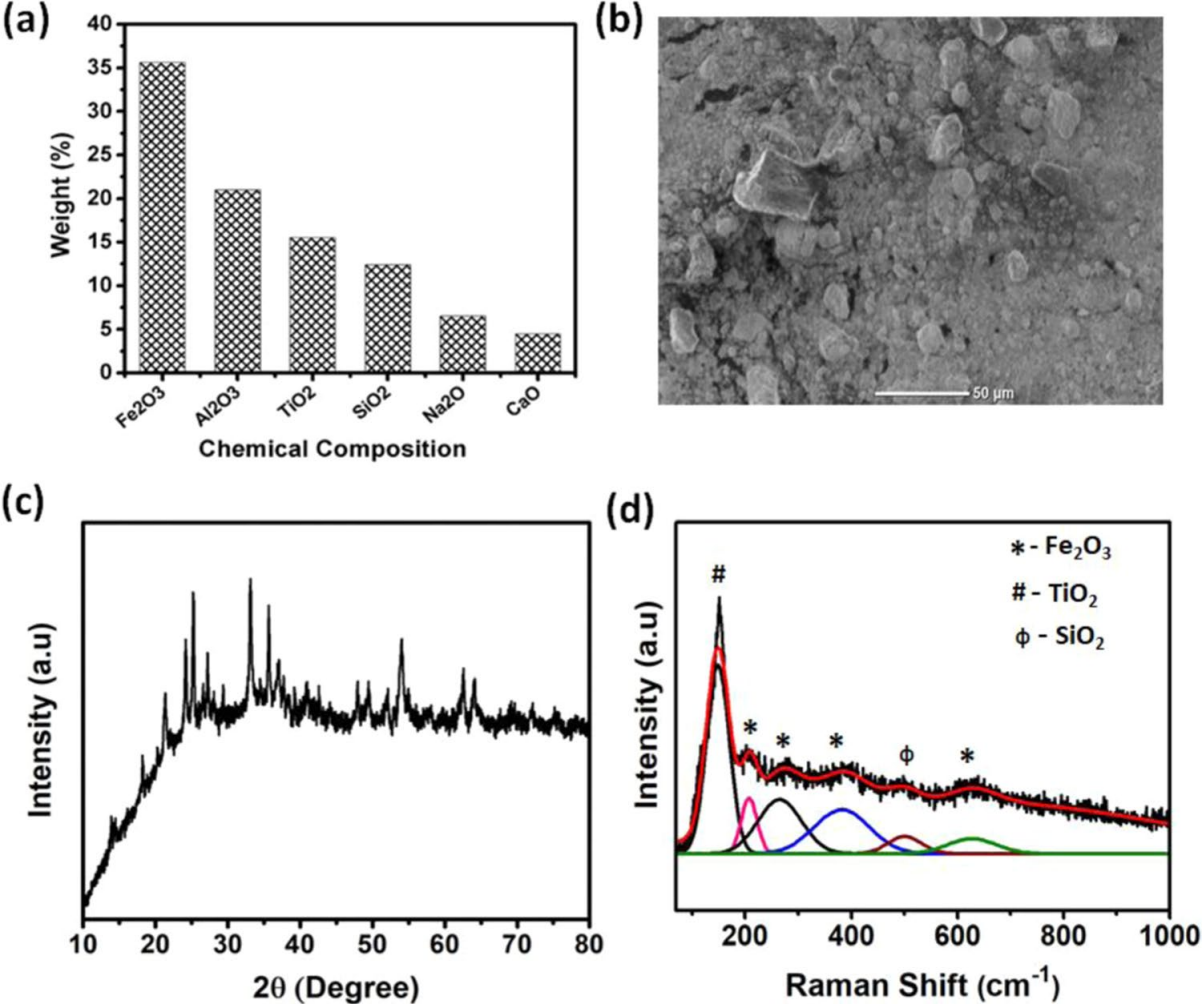
(**a**) Chemical composition, (**b**) SEM image (**c**) XRD and (**d**) Raman spectra of red mud.

The red mud was characterized for chemical analysis and their chemical constituent present in Fig. 2(a). From this figure, it is shown that the major metal oxides in red mud are Fe_2_O_3_, Al_2_O_3_, TiO_2_, SiO_2_ and CaCO_3_ and minor elements are Na, K, Cr, V, Ni, Ba, Cu, Mn, Pb, Zn, etc. Figure 2(b) shows the morphology of red mud which reveals that the red mud particles are irregular in shape with the size of ~20 µm. The phase and chemical constituent present in red mud were analyzed using X-ray diffraction pattern as shown in Fig. 2(c). The red mud exhibits the presence of hematite (Fe_2_O_3_), alumina (Al_2_O_3_), gibbsite (Al (OH)_3_) quartz (SiO_2_), anatase and rutile (TiO_2_) and calcite (CaCO_3_)^30^. The Raman results for red mud are shown in Fig. 2(d). The various broad peaks at the same wavenumber are responsible for different metal oxides in red mud. Due to a large amount of hematite (Fe_2_O_3_) in red mud, four dominating peaks at 211, 275, 387, 620 cm^−1^ are shows in this spectra. The peak at 150 and 508 cm^−1^ are due to anatase (TiO_2_) and quartz (SiO_2_) respectively^36^.

**Figure 3:**
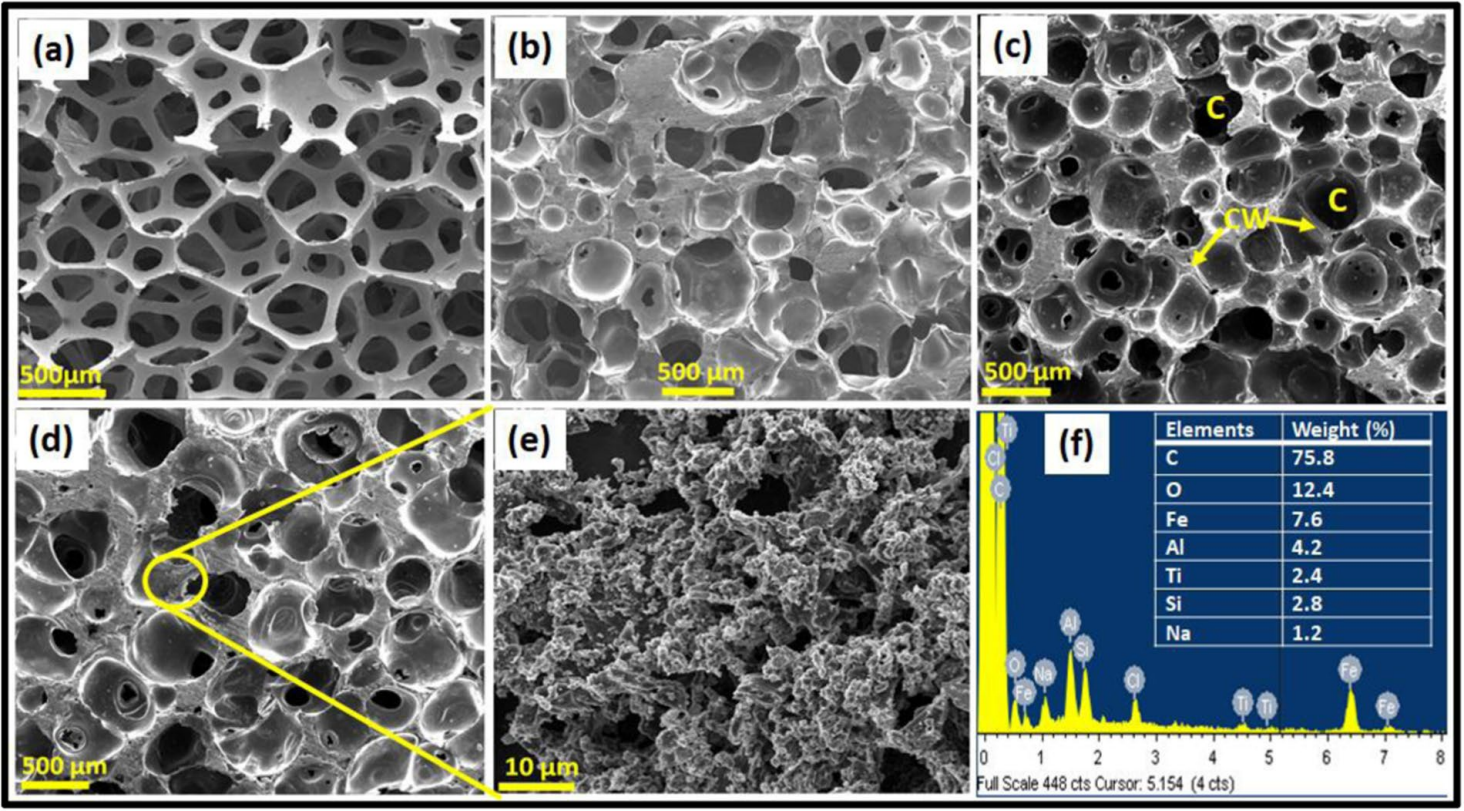
SEM images of (**a**) PU foam, (**b**) CF, (**c**) CF-RM10 (**d)** CF-RM20, (**e**) magnified image of CF-RM20 showing the distribution of red mud over the surface of carbon foam, (**f**) EDS analysis of CF-RM20 which showing the elements presents in carbon-red mud hybrid foam.

Figure 3(a–e) shows the open cell structure of carbon-red mud hybrid foams. The SEM image of PU foam (Fig. 3(a)) shows that all the pores are elliptical with the size range of 350–450 µm. The SEM image of CF indicates that the pores are not exactly spherical or elliptical but near to spherical (Fig. 3b). It is also found that the pore size displays a broad range of 200–300 µm which could be due to shrinkage in phenolic resin impregnated PU foam during carbonization. Some pores are broken during sample preparation. Figure 3(c,d) shows the morphology of the CF-RM10 and CF-RM20 respectively; it can be seen that the red mud has been uniformly and densely deposited on the cell wall of the foam.

From Fig. 3(d), it is also observed that on increasing the loading of red mud contents in carbon foam, the microporosity created by red mud has increased. Due to this microporosity, CF-RM20 may have lower compressive strength. The magnified image of CF-RM20 is shown in Fig. 3(e). In this image, red mud clustering formation is also observed in some places which could be responsible for the decreased open porosity in carbon foam. Figure 3(f) shows the EDS analysis of CF-RM20. The EDS at the cell wall of the carbon-red mud hybrid foam shows that the major elements are C, O, Fe, Al, Ti, Si, and Na.

**Figure 4:**
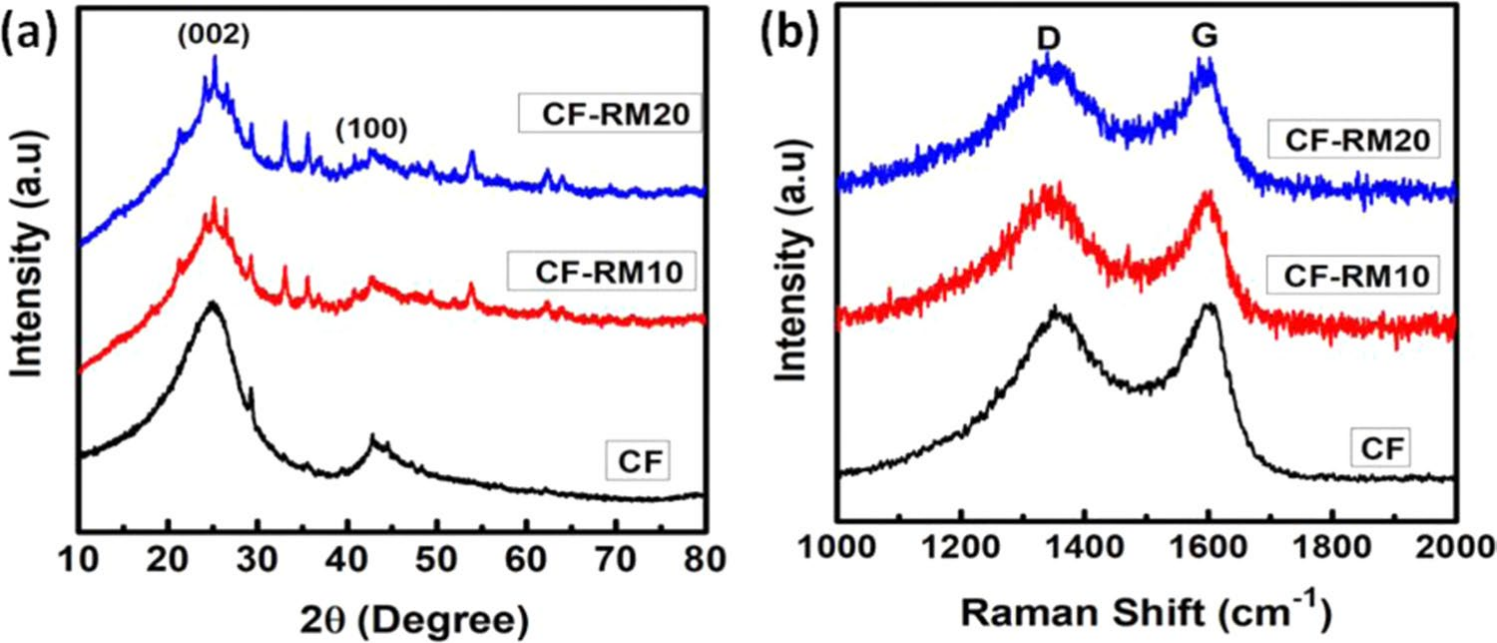
(**a**) XRD and (**b**) Raman spectra of carbon-red mud hybrid foams.

The structural analysis of carbon-red mud hybrid foams has been characterized by an X-ray diffraction pattern and shown in Fig. 4(a). The pristine CF shows two diffraction peaks which are assigned to (002) and (100) lattice planes of hexagonal graphite. In CF, a broad diffraction peak (002) at 24.90, corresponding to a d-spacing of 0.3574 nm, indicates the nature of amorphous carbon. The peaks present in the XRD pattern of red mud (Fig. 2(c)) have also been observed in all the carbon-red mud hybrid foams indicating the incorporation of red mud in the carbon matrix. When red mud was incorporated in the CF, the 002 peak in CF-RM20 gets slightly narrow and peak shifts to a higher 2θ angle which could be due to the incorporation of red mud (multiphase component) in the carbon matrix. The d-spacing of CF-RM20 decreased to 0.3518 nm for 20% loading of the red mud.

Raman spectroscopy was employed to gain more insight into the structural differences in carbon-red mud hybrid foams as shown in Fig. 4(b). In general resin-based CF exhibits two peaks at 1580 and 1350 cm^−1^. The peak at about 1580 cm^−1^ (G -band), corresponding to an E_2g_ mode of graphitic carbon, is related to the vibration of the sp^2^ bonded carbon atoms in a two-dimensional hexagonal lattice, whereas the peak at about 1325 cm^−1^ (D band) is related to the amorphous carbon species, defects and disorder in the hexagonal graphitic layers^37^. After the incorporation of red mud in carbon foam, the intensity of D peaks slightly increases in CF-RM10 and CF-RM20. The high intensity of D peaks in CF-RM10 and CF-RM20 indicate the removal of carbon atoms in the lattice which leads to higher defect density and obvious broadening of G peak. The intensity ratio of D band to G band (I_D_/I_G_) is also used to calculate the degree of disorder in the graphitic structure. The value of I_D_/I_G_ of as-received CF is observed to be 0.9347 however, it increases to 1.183 when 20% red mud loaded in carbon foam (CF-RM20). The results indicate that as the loading of red mud increases the density of defects gradually increases due to red mud induced distortion of the carbon atoms in the lattice.

**Figure 5:**
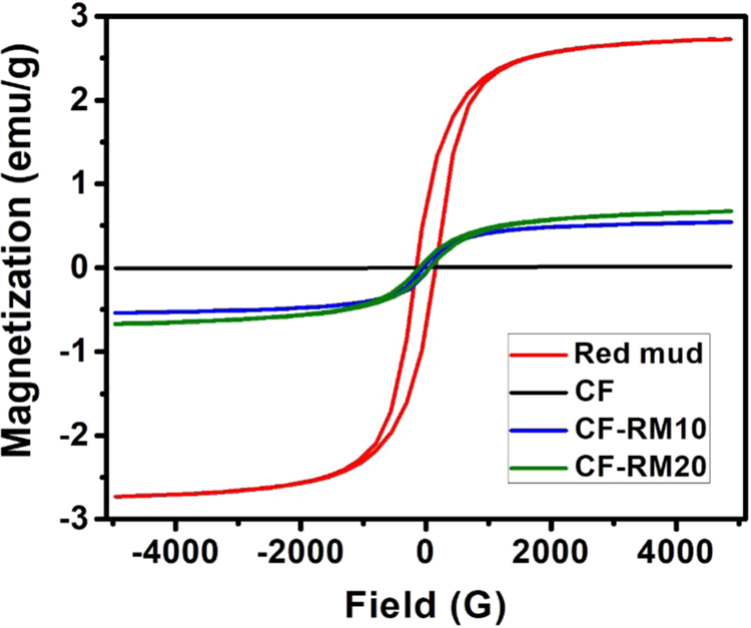
Magnetic hysteresis loops of red mud, CF, CF-RM10 and CF-RM20 at room temperature.

The magnetic dipoles are one of the key requirements in designing materials that can attenuate EM radiations. The field dependence of magnetization for red mud, CF, CF-RM10, and CF-RM20 was examined by vibrating sample magnetometer (VSM) at room temperature. The hysteresis loops for all the samples are shown in Fig. 5. The saturation magnetization of red mud is found to be 2.73 emu/g due to the presence of a high amount of hematite (Fe_2_O_3_) in the red mud. As expected, CF does not show the magnetization. However, the magnetization value for CF-RM10 and CF-RM20 are found to be 0.54 and 0.67 emu/g respectively. These results suggest that the loading of red mud in carbon foam increases the magnetization due to increased amount of magnetic particles.

The physical, mechanical and electrical properties such as density, porosity, compressive strength, electrical conductivity and EMI shielding of carbon-red mud hybrid foams with an increasing amount of red mud contents are shown in Table 1. The density of carbon foam is directly related to the red mud content and sintering temperature. Pristine CF shows the density of about 0.30 g/cm^3^ at sintering temperature 1100 °C. The density of carbon foams continuously increases with increasing red mud content and reaches maximum to 0.46 in CF-RM20. This is attributed to the high density of red mud (2.7 g/cm^3^) as compared to CF. Also, red mud has multiphase components (metal oxides) which tend to increase its density by sintering at high temperatures (1100 °C). The open porosity for carbon-red mud hybrid foams is given in Table 1 which is measured to be 85, 83, 82, 80 and 78 in CF, CF-RM5, CF-RM10, CF-RM15, and CF-RM20, respectively.

**Table 1:** Illustrates the average values of density, open porosity, compressive strength, electrical conductivity and EMI shielding effectiveness of carbon-red mud hybrid foams.

Samples	Density (g/cm^3^)	Open Porosity (%)	Compressive Strength (MPa)	Electrical Conductivity (S/cm)	EMI SE(dB) in the range of 8.2–12.4 GHz
CF	0.30	85.0	4.8	35.4	20.8
CF-RM5	0.35	83.0	5.8	29.0	30.3
CF-RM10	0.38	82.0	6.6	24.6	34.5
CF-RM15	0.42	80.0	7.8	18.0	39.6
CF-RM20	0.46	78.0	6.5	14.8	49.0

The stress-strain curve is used to analyze the mechanical properties of carbon foam with different red mud contents, and results are given in Table 1. All the carbon-red mud hybrid foams samples were tested at 80% compressive strain. It is well known that the strength of the foam materials depends on many factors such as density, microstructure, and reinforcing materials. The red mud has high density therefore by increasing red mud contents, the density of foam increases continuously which is responsible for increased compressive strength. The foam structure is described using cell wall thickness and length of the cell edge, which have the following relation with density^38^.

1 $${\left(\frac{t}{l}\right)}^{2}=\frac{{{\rho }}_{f}}{{{\rho }}_{d}}$$

**Figure 6:**
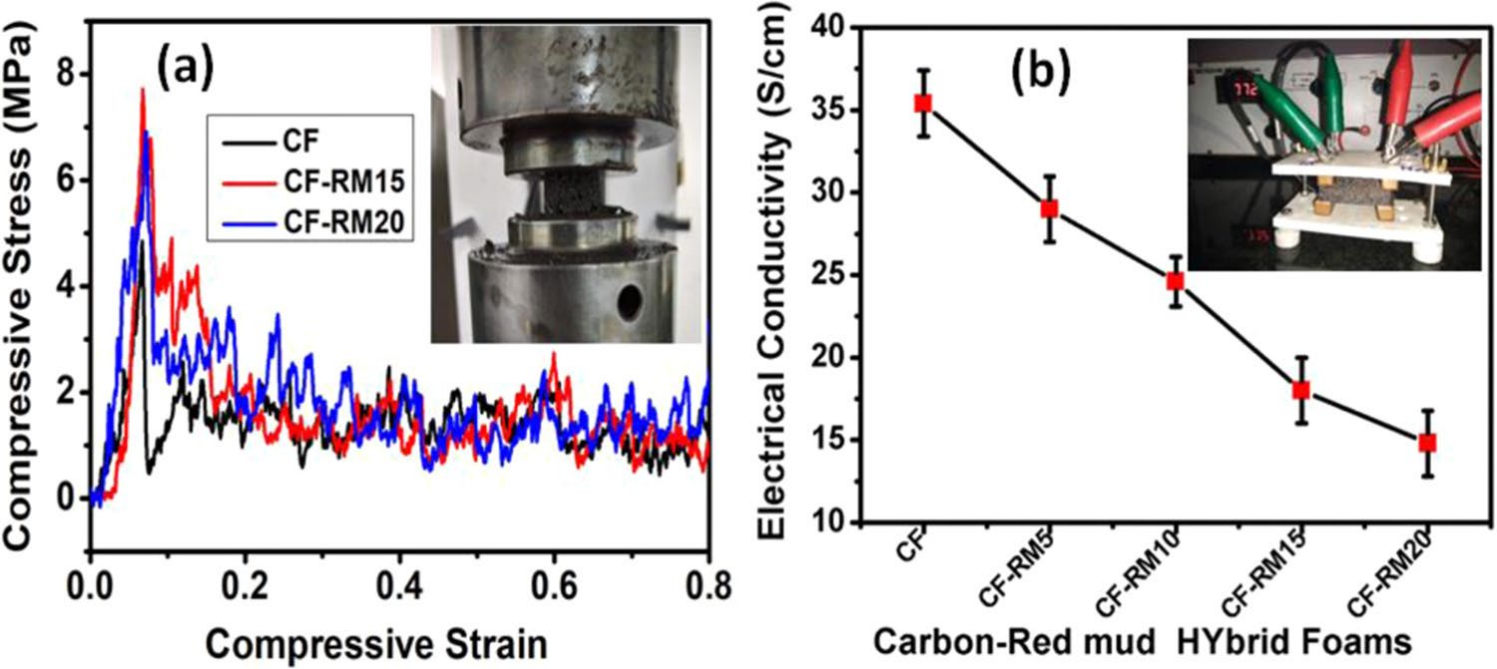
(**a**) Compressive strength and (**b**) electrical conductivity of carbon-red mud hybrid foams.

Where t is the thickness of the cell wall, l is the length of the cell edge, ${\rho }_{f}$ is the bulk density of foam and ${\rho }_{d}\,$is the real density of the foam. As the real density is considered to be constant, the foam with higher bulk density, which implies a thicker cell wall and shorter cell edge, has higher compressive strength. It is seen from Fig. 6(a), the compressive strength of CF is about 4.8 MPa which first increased to 7.8 MPa with an increase in 15% red mud content in CF-RM15 and then decreased with further increase in red mud content in CF-RM20 and shows higher compressive strength than CF because the red mud particles stop the cracks propagation in foam samples. However, when the content of red mud exceeds 15%, the red mud may form the clusters. As a consequence of this, the load transfer from carbon to the red mud was not efficiently accomplished, leading to a lower compressive strength in CF-RM20 than CF-RM15.

The electrical conductivity of carbon-red mud hybrid foams having different red mud loading was measured at room temperature and results are plotted in Fig. 6(b). The electrical conductivity of CF is measured to be 35.4 S/cm. The electrical conductivity of carbon-red mud hybrid foams decreases with an increasing weight percentage of red mud and measured to be 29.0, 24.6, 18.0 and 14.8 in CF-RM5, CF-RM10, CF-RM15, and CF-RM20, respectively. The decrease in electrical conductivity with an increase in the loading content of red mud in hybrid foams is obvious because of the dielectric, and insulating nature of the red mud. However, the magnetic nature of red mud enhances the permeability of carbon foams, leading to magnetic loss. Moreover, the incorporation of red mud powder provides new interfaces for the accumulation of virtual charges, which leads to interfacial polarization. Therefore, it may be concluded that the balance of electric and magnetic properties is very important to achieve high absorption in carbon-red mud hybrid foams.

**Figure 7:**
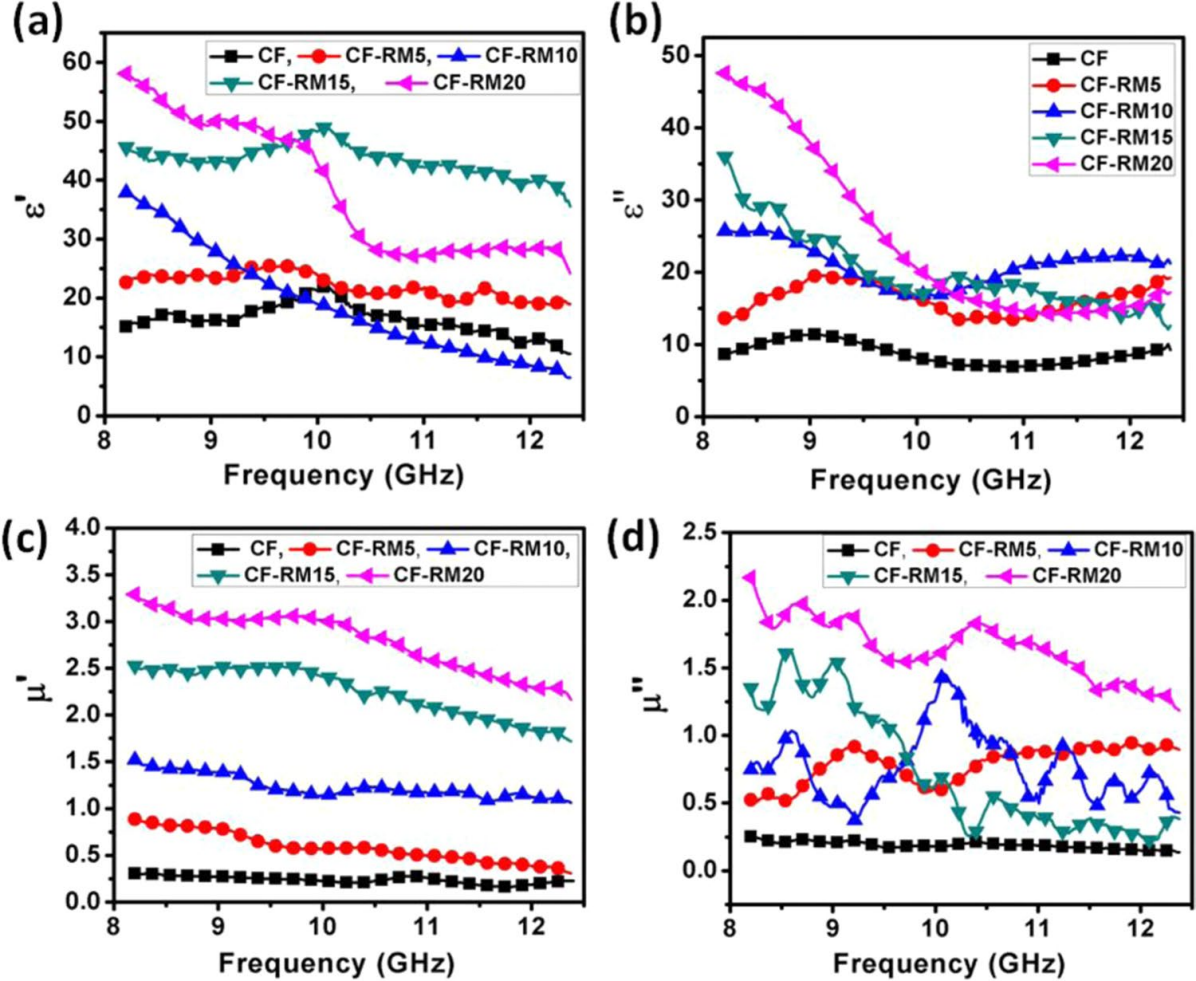
(**a**) real permittivity (ε′), (**b**) imaginary permittivity (ε″), (**c**) real permeability (µ′) and (**d**) imaginary permeability (µ˝) of carbon- red mud hybrid foams in the frequency range of 8.2 to 12.4 GHz.

The electromagnetic parameters i.e. relative complex permittivity (ε′ and ε″) and relative complex permeability (µ′ and µ″) of carbon-red mud hybrid foams were measured at room temperature using vector network analyzer in X band (8.2–12.4 GHz) frequency region and values are presented in Fig. 7(a–d).

The Nicholson–Ross and Weir theoretical calculation were used to determining these complex parameters with frequency using scattering parameters (S11 and S21)^39,40^. In a dielectric material, the parameter ε´ is the real part of the permittivity which reflects the storage capacity of the electromagnetic energy, while the ε″ is the imaginary part of the permittivity which represents the energy dissipation processes including the conduction and polarization relaxation loss. Similarly, real permeability (µ′) and imaginary permeability (µ″) are magnetic storage and magnetic losses, respectively. The values of ε′ and ε″ for carbon-red mud hybrid foams are presented in Fig. 7(a,b). In case of CF, the ε′ and ε″ are observed 14.8 and 8.5 respectively at 8.2 GHz. Both the values of ε′ and ε″ of carbon-red mud hybrid foams increase with increasing loading of red mud. The highest value of ε′ and ε″ in CF-RM20 is found to be 58.6 and 47.6 at a frequency of 8.2 GHz, respectively. The increase in complex permittivity with an increase in red mud content within the carbon foam can be ascribed to enhanced dipole orientation polarization and interfacial polarization, considering the other mechanisms such as electronic and ionic polarization. It is known that the red mud has multiphase elements (metal oxides) which create interfacial polarizations between metal oxides and carbon atoms^41^. Additionally, these metal oxides create defects in carbon structure and work as a polarizing center, resulting in high permittivity in carbon-mud hybrid foams. For better understanding, the value of ε′ and ε″ can be described by Debye theory^41^.

2 $${\rm{\varepsilon }}{\prime} ={\varepsilon }_{\infty }+\frac{{\varepsilon }_{s}-\,{\varepsilon }_{\infty }}{\,1+{\omega }^{2}{\tau }^{2}}$$

3 $${\rm{\varepsilon }}{\prime\prime} =\frac{{\varepsilon }_{s}-{\varepsilon }_{\infty }}{1+{\omega }^{2}{\tau }^{2}}\omega \tau +\frac{\sigma }{\omega {\varepsilon }_{s}}$$

Here ε_s_ is the static permittivity, ε_∞_ represents optical dielectric constant, ω defines the angular frequency, τ is the polarization relaxation time and σ is the electrical conductivity. From Eq. 3, it is observed that the values of ε″ are lower than ε′ in carbon-red mud hybrid foams due to decreased electrical conductivity of carbon-red mud hybrid foams. On the other hand, the increase of ε″ in CF-RM20 as compared to CF might be due to orientation and space charge polarization which are responsible for improving absorption in carbon-red mud hybrid foam.

Further improvement in the absorption component of carbon-red mud hybrid foams can also be correlated with complex permeability (µ′ and µ″) and their values are depicted in Fig. 7(c,d). The real permeability (µ′) of the carbon-red mud hybrid foams increases with increase in contents of red mud, and the values are found to be 0.30, 0.90, 1.52, 2.54 and 3.31 for CF, CF-RM5, CF-RM10, CF-RM15, and CF-RM20, respectively at a fixed frequency of 8.2 GHz. At the same time, the imaginary part of permeability (µ″) of these carbon-red mud hybrid foams also follows the same trend and their values are 0.25, 0.53, 0.70, 1.4 and 2.2 for CF, CF-RM5, CF-RM10, CF-RM15, and CF-RM20, respectively. This trend is due to the presence of Fe_2_O_3_ in red mud which improves the magnetic properties of carbon foam. It is also observed that the total loss (dielectric and magnetic loss) of carbon-red mud hybrid foams increases with an increase in red mud content which is ascribed to their enhanced magnetic properties. Herein, the magnetic loss in carbon-red mud hybrid foam is due to eddy current loss, hysteresis loss, natural resonance and anisotropic filed effects which all contribute to enhancement in the absorption component.

The EMI shielding effectiveness is defined as the logarithmic ratio of incident power (P_I_) and transmitted power (P_T_) which is depends on three components i.e. reflection (R), absorption (A) and multiple reflections (M).

4 $${{\rm{SE}}}_{{\rm{T}}}=10{\log }_{10}\left(\frac{{P}_{I}}{{P}_{T}}\right)={{\rm{SE}}}_{{\rm{A}}}+{{\rm{SE}}}_{{\rm{R}}}+{{\rm{SE}}}_{{\rm{M}}}$$

Multiple reflections (SE_M_) are the internal reflections between the internal surface of the shield material. In a particular case where the shielding is higher than 10 dB, the multiple reflections can be neglected and the total EMI shielding values (SE_T_) can be calculated by following equation^42–44^.

5 $${{\rm{SE}}}_{{\rm{T}}}={{\rm{SE}}}_{{\rm{A}}}+{{\rm{SE}}}_{{\rm{R}}}$$

6 $${\rm{T}}={|{S}_{12}|}^{2}={|{S}_{21}|}^{2}$$

7 $${\rm{R}}={|{{\rm{S}}}_{11}|}^{2}={|{{\rm{S}}}_{22}|}^{2}$$

8 $${\rm{A}}=1-{\rm{R}}-{\rm{T}}$$

where S_11_ is a forward reflection coefficient, S_22_ is a reverse reflection coefficient, S_12_ represents a forward transmission coefficient, S_21_ is a reverse transmission coefficient. Further, after primary reflection, the intensity of incident EM wave inside the shield materials is based on (1-R), which can be used to calculate effective absorbance (A_eff_);

9 $${{\rm{A}}}_{eff}=\frac{(1-{\rm{R}}-{\rm{T}})}{(1-{\rm{R}})}$$

therefore, SE_A_ and SE_R_ can be calculated using the following equations.

10 $${{\rm{SE}}}_{{\rm{A}}}({\rm{dB}})=10{\log }_{10}(1-{{\rm{A}}}_{{\rm{eff}}})=10{\log }_{10}\,\left(\frac{{\rm{T}}}{1-{\rm{R}}}\right)$$

11 $${{\rm{SE}}}_{{\rm{R}}}({\rm{dB}})=10{\log }_{10}(1-{\rm{R}})$$

**Figure 8:**
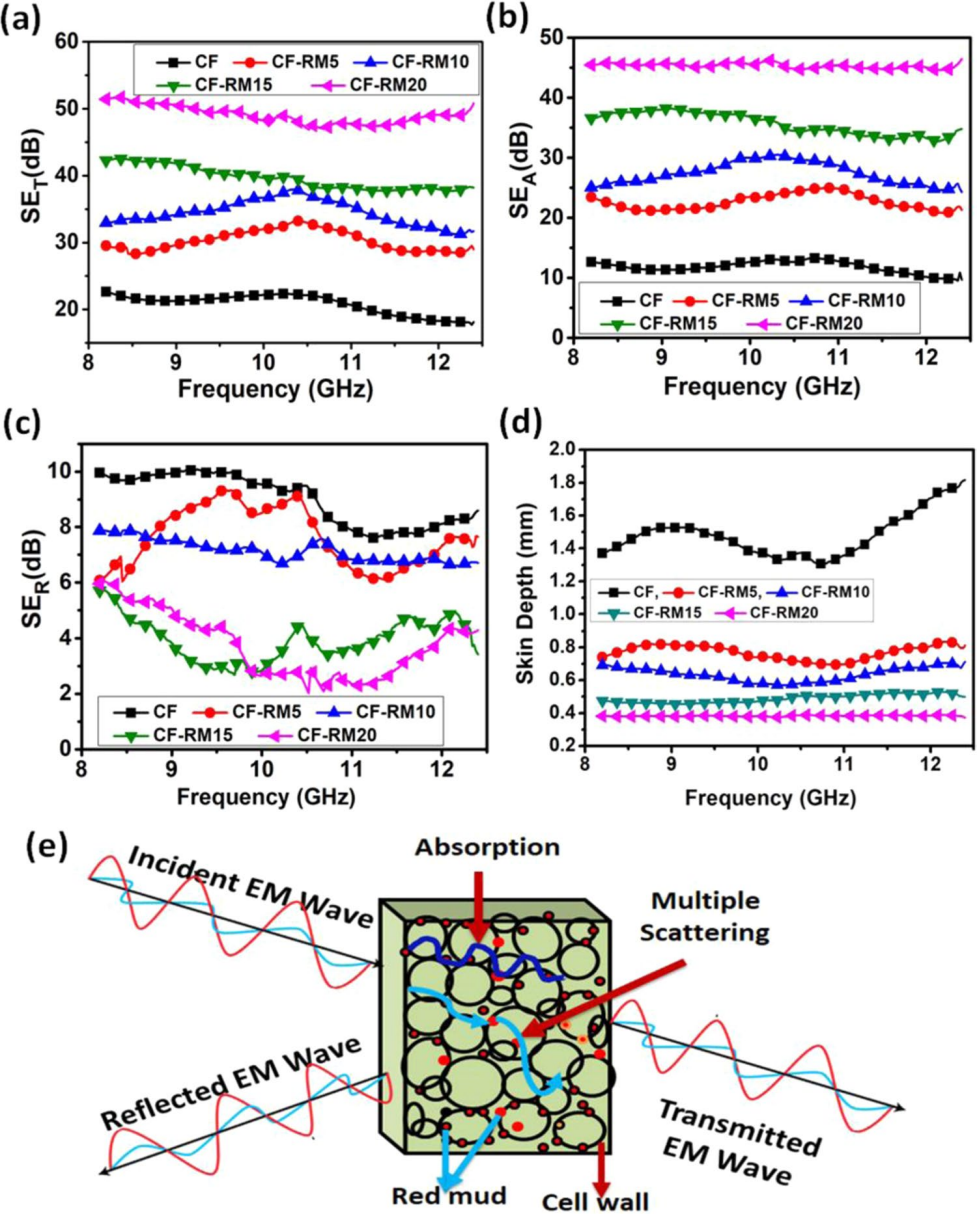
EMI shielding; (**a**) Total (SE_T_), (**b**) Absorption (SE_A_), (**c**) Reflection (SE_R_), (**d**) skin depth of carbon-red mud hybrid foams in the frequency range of 8.2 to 12.4 GHz and (**e**) schematic representation of EMI shielding mechanism in carbon-red mud hybrid foam.

Figure 8(a–c) shows the variation in absorption, reflection and the total EMI shielding of the carbon-red mud hybrid foams in the X-band frequency range (8.2–12.4 GHz). It is shown in Fig. 8(a) that SE_T_ value for CF is observed to be 22.6 dB at a frequency of 8.2 GHz that is shared by reflection component (9.9 dB) as well as absorption component (12.7 dB). Initially, the EMI SE_T_ value of CF-RM5 is increased to 29.5 dB and SE due to SE_A_ and SE_R_ are found to be 23.5 dB and 6.0 dB, respectively at a frequency of 8.2 GHz. Further addition of red mud (up to 20%) further increases the SE_T_ value to 51.4 dB for CF-RM20 with a much higher value of SE_A_ (45.5) and lower value of SE_R_ (5.9) at 8.2 GHz frequency. The observed value of SE_T_ for CF-RM20 is found to be maximum among all the carbon-red mud hybrid foams. The high value of SE_A_ compared to SE_R_ confirms that the shielding of EM waves is mainly dominated by the absorption component. The high value of SE_T_ in CF-RM20 may be due to the high dielectric and magnetic loss, which is also well supported by complex permittivity and permeability. Moreover, the non-conducting nature of red mud gives rise to interfacial polarization, eddy current loss and magnetic loss due to spin resonance, magnetic domain movement and relaxation of the magnetization. Furthermore, the porous, interconnected network structure and high surface area of carbon-red mud hybrid foam provide much better EM wave absorption activity^45^ which offers a greater accumulation of charge at the carbon-red mud interface, hence an increased absorption is observed.

The enhancement in the absorption of carbon-red mud hybrid foams can further be correlated with skin depth. The skin depth (δ) is defined as the distance at which the energy of incident EM wave decreases to 1/e (37%), which is represented by the following equation^46^;

12 $$\delta ={\left(\frac{2}{\omega \mu \sigma }\right)}^{1/2}=-8.68\left(\frac{t}{{{\rm{SE}}}_{{\rm{A}}}}\right)$$

where (ω) is the angular frequency, $\sigma \,$is the electrical conductivity ${\rm{\mu }}\,$is the magnetic permeability and t is the thickness of the material. Figure 8(d) shows the variation of skin depth in carbon-red mud hybrid foams at the frequency range 8.2–12.4 GHz. It can be seen that the skin depth of carbon-red mud hybrid foams decreases with an increase in the loading of red mud. The skin depth of CF is found to be 1.36 mm whereas in CFRM20 the skin depth reduces to 0.38 mm at 8.2 GHz. The decrease in the skin depth with increasing loading of red mud is responsible for the enhancement in absorption.

In addition to that EM wave by shielding materials is related to electrical conductivity and magnetic permeability as^47^

13 $${{\rm{SE}}}_{{\rm{A}}}({\rm{dB}})=8.68\,t{\left(\frac{\sigma \omega {\mu }_{r}}{2}\right)}^{1/2}$$

where, t, σ, ω and μ represent thickness, conductivity, angular frequency and relative permeability of the shield material respectively. As mentioned above, the electrical and magnetic properties play an important role in improving the SE_A_. From Eq. (13) it is found that SE_A_ is directly related to permeability (µ), a slight increase in permeability (magnetic loss) causes excellent absorption which is also responsible for superior EMI shielding performance in carbon-red mud hybrid foam. The interaction mechanism of electromagnetic wave with carbon-red mud hybrid foam is also described in Fig. 8(e). Therefore, it can be concluded that the red mud has some synergetic effect on the EMI shielding properties of the carbon foam; thus, carbon-red mud hybrid foam holds great promise in EMI shielding applications.

**Figure 9:**
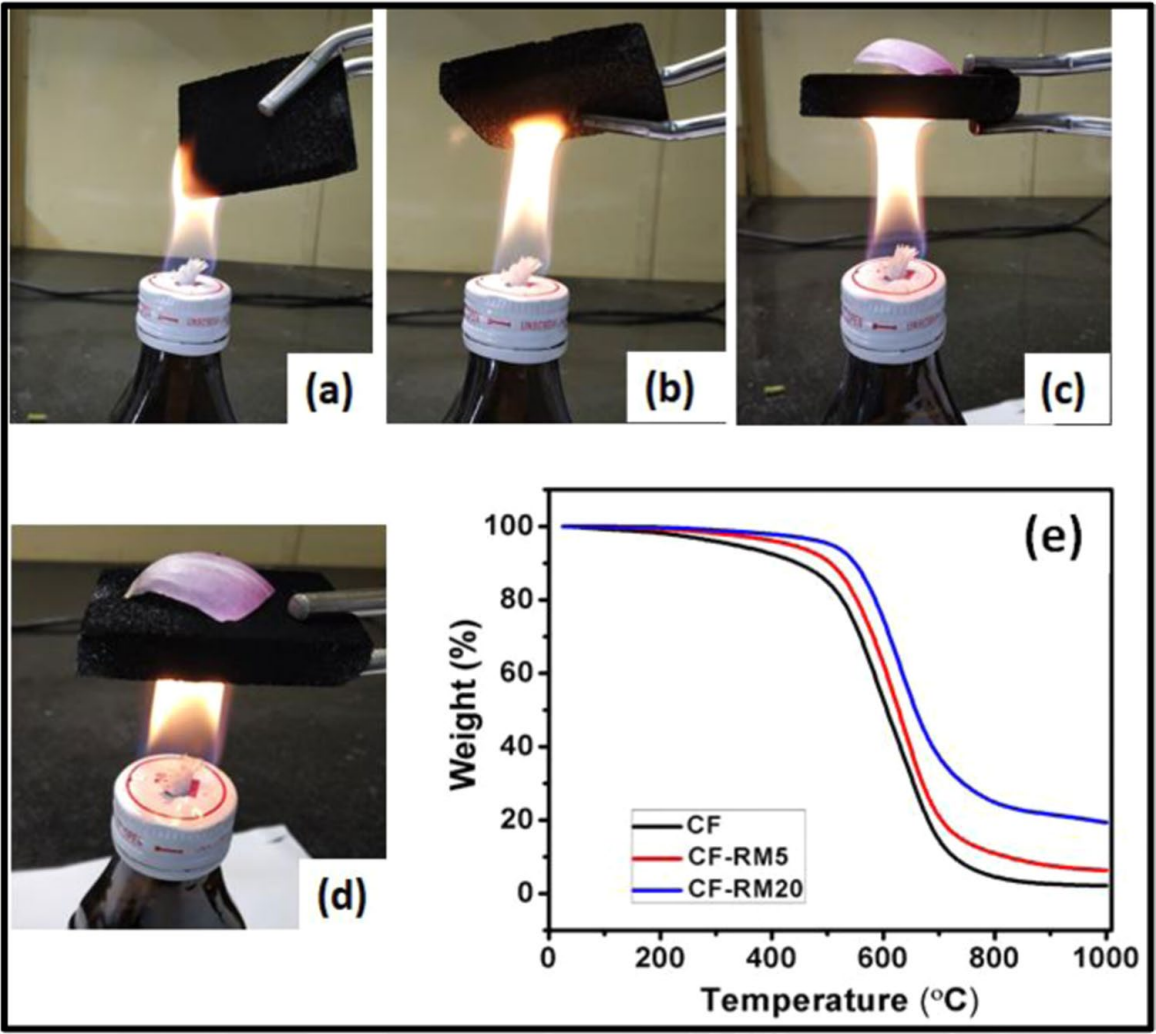
Fire-resistant performance for different periods: (**a**) 10 s, (**b**) 20 s, (**c**) 30 s, (**d**) 60 s and (**e**) thermal stability of carbon-red mud hybrid foams.

The fire-resistant and high thermal stability of carbon foam is an important factor in EMI shielding applications because absorbed radiation may damage the structure of foam^48^. The carbon-red mud hybrid foam exhibited excellent fire resistance for a long time as shown in Fig. 9(a–d). The flame test experiment of carbon-red mud hybrid foam was performed on an alcohol burner in which the foam sample was ignited on the flame for a maximum time of 60 seconds. Figure 9(a–d) displays that CF-RM20 has excellent fire-resistant performance due to the thermal insulating nature of metal oxides present in red mud. This is reflected by maintained shape of CF-RM20 foam after ignition on the outer flame of the alcohol burner.

To further confirm the thermal stability of carbon-red mud hybrid foams, a thermal gravimetric analysis (TGA) test was performed at 1000 °C under air^35^. Figure 9(e) shows the TGA curve of carbon-red mud hybrid foams with different loading of red mud contents. The results show that CF shows 4% weight loss at 300 °C, however, CF-RM5 and CF-RM20 show 2 and 1% weight loss respectively at the same temperature. The results also reveal that CF shows weight loss in two steps. At first, it shows very minor weight loss (about 4.0%) which is observed at the temperature of 300 °C. However, in later step major weight loss (83.0%) takes place between the temperature range of 400- 700 °C. The final major weight loss step corresponds to the complete pyrolysis of carbon foam fragments into a smaller fraction and gaseous by-products. Whereas 5% and 20% loading of red mud in carbon foam change the thermal degradation steps and the minor weight loss of 4% and 2% was observed at 400 °C for CF-RM5 and CF-RM20 respectively. In both the samples (CF-RM5 and CF-RM20) major weight loss takes place between the temperature range of 500–800 °C. These show that thermal decomposition temperature increases with increasing the loading of red mud and indicating that the carbon-red mud hybrid foams possess excellent thermal stability. This is attributed to multi-phase metal oxides (Fe_2_O_3_, Al_2_O_3_, SiO_2_, TiO_2,_ etc.) present in red mud which are thermally stable up to ~1500 °C. Additionally, as evident from the TG traces, with the increase of red mud content, the corresponding ash residue also increases from 2.2% (CF) to 19.4% (CF-RM20) which indicates the presence of metal oxides inside the carbon-red mud hybrid foams.

## Conclusions

The carbon-red mud hybrid foams with superior EMI shielding and excellent fire-resistant properties were fabricated via PU foam template route. These carbon-red mud hybrid foams have been explored for EMI shielding in the X-band (8.2–12.4 GHz) region, fire-resistant in alcohol flame and thermal stability up to 1000 °C in an oxidative environment. By incorporation of the red mud in the carbon matrix not only improve the EMI shielding properties but also improve the flame resistance and thermal stability. The CF-RM20 possesses high dielectric and magnetic properties with improved EMI shielding of 51.4 dB at 8.2 GHz, which is 127% higher than CF. Further, the incorporation of red mud content in carbon foam provides interfacial polarization, eddy current loss, and magnetic loss and open-cell interconnected networks which give rise to the high surface area and help to improve the absorption of 250% in CF-RM20. The present investigation offers a new way to fabricate carbon-red mud hybrid foams by utilizing red mud (industrials waste) and can be used as effective shielding and fire-resistant materials for defense and aerospace applications.
